# Medical Opinion and Movement

**Published:** 1909-01-02

**Authors:** 


### Medical Opinion and Movement.

OBSERVATIONS on the effect of drugs on the digestive tract by means of the $x$-rays continue to accumulate. It will be remembered that the passage of the food is made visible by mixing bismuth with it. Dr. R. Magnus has in this way studied the specific action of infusions of senna leaves and of castor oil. He finds that the infusion of senna leaves has no influence upon the movements of the stomach or small intestine, but as soon as the food reaches the large intestine the purgative effect is apparent. The increased peristalsis is found to be the same in dogs after the spinal cord has been destroyed from the eleventh dorsal segment, from which it is argued that the drug does not act through the nervous system, but directly upon the wall of the intestine. On the other hand, castor oil increases the peristalsis of the whole of the intestinal tract as well as the stomach. The oily factor of the purgative is said partly to counterbalance this effect on the stomach, but when the active principle of the oil, the ricinolic acid, is administered pure, the increase in peristalsis of the stomach is clearly demonstrated. The action is most pronounced in the small intestine. On the other hand, the large intestine is only slightly affected, and it is only under the pressure of the material from the small intestine that the large intestine eventually evacuates its contents.

---

IT has been long taught in text-books that gall-stones are very much less common in Asiatics than among Europeans. Professor Leonard Rogers, feeling very doubtful of this assertion, which has been made by the highest authorities on the subject in this country, has investigated the post-mortem records of Calcutta for a long series of years, and has published the results in the *Indian Medical Gazette*. He finds that gall-stones are quite as common in Calcutta as in England, if not more so; that they are twice as common there in females as in males, both among Hindus and Europeans, and in all cases independently both of sedentary habits and of tight lacing. In all races they are rare under the age of thirty, and increase steadily in frequency with advancing years. After making due allowance for age and sex, Mahomedans are less liable than Hindus, and Europeans more so. Nevertheless, these racial differences do not appear to be fully explained by the commonly accepted theory that a carbohydrate diet favours gall-stones more than a nitrogenous one, for this factor should produce a lower incidence among Europeans. Another peculiar fact elicited is the close association of cirrhosis of the liver and of granular kidney with gall-stones. The former is a much commoner disease in Calcutta than in England, for 5 per cent. of the 5,000 cases under examination presented it. Indeed, some form of vascular or degenerative change, most often fatty, is said to be the rule rather than the exception in Calcutta. Allowing for this, it is still somewhat astonishing to find that amongst gall-stone cases 18.2 per cent. had also hepatic cirrhosis, and that in three-fourths of these the lesions were advanced in degree. But even more striking are the figures for granular fibrotic disease of the kidney, which was found in 40 per cent. of the gall-stone cases. Professor Rogers believes that it is these renal and hepatic diseases which dispose to gall-stones by causing metabolic changes, which may easily be conceived to alter the constitution and consistence of bile in such a manner as to favour the formation of calculi.

---

INJURIES to the bladder during the radical cure of an ordinary hernia are now recognised as less uncommon than was formerly supposed. Attention was first emphatically called to this complication by Bruner in 1898, and subsequently by Moynihan and others; and the subject was again discussed by Skeel at the recent meeting of the American Association of Gynæcologists. This observer concludes with the suggestion that for a day or two before operation for radical cure methylene blue should be administered to the patient as a matter of routine, in order that any perforating injury or threatened injury to the bladder may be recognised at once. He points out that the wall of the bladder, so far from being muscular and easy to recognise in these cases, is very thin and may be cut or torn even by the most experienced. The mortality of cases in which this accident happens is said to be 22 to 25 per cent., judging from published statistics; but the author believes that the publication of all cases would show a higher mortality still. Inasmuch as the mortality of radical cure itself is practically nil, it is evident that the avoidance of the bladder is a part of the technique in which advance is most essential. Skeel accepts Bruner's classification of bladder hernias into two kinds. First that in which there is a true hernia of part of the bladder, or of a diverticulum from it. This is very uncommon. Secondly that in which traction on the hernial sac drags down into the operation field the reflection of the peritoneum from the bladder to the pelvic wall and along with it a portion of the bladder itself. He believes this second sort vastly to outnumber the first, and emphasises the greatly increased proportion of cases noticed and recorded since attention was drawn to the matter by the observers already quoted.

---

MISTLETOE (*viscum album*) is an old remedy, long fallen into disuse, but recently revived. Its chief action lies in the direction of lowering the systemic blood-pressure, and it has been found to be of particular benefit in cases of arteriosclerosis. It has also been employed in cases of cerebral hæmorrhage, phthisical hæmoptysis, severe epistaxis, and the like, but as a remedy in such acute conditions it is likely to have but secondary rank. It has been found that the active principle rapidly disappears when the mistletoe is dried. The therapeutic action is only obtained when preparations are made from the fresh plant. An aqueous extract of the mistletoe is the form in which it is chiefly recommended by R. Gaultier, in the form of pills, the dosage being such that the patient receives about three grains of the extract daily.

Spengler, of Davos, has a theory that the source of immunity producing substances in tuberculosis is the red blood cells, the leucocytes acting as secondary storing stations only. Under his auspices a therapeutic preparation (J.K.) has been made, which contains not only the immune substances of tuberculosis, but also those of the pyogenic organisms, thus fulfilling for phthisis the indication often advanced that secondary septic infections should be attacked as well as the primary tuberculous one. Two reports on this preparation are now to hand. Szabóky (*Orvosi Hetilap* No. 28) has found that cases treated by this means alone do not do so well as those undergoing the usual hygienic regimen. He thinks, however, that Spengler's inoculation might prove a useful adjuvant. Torday's (*Budapesti Orvosi Ujság* No. 30) cases made no clinical progress that could be reckoned at all exceptional. These two reports rather bear out Pottenger's dictum, that in tuberculosis any treatment not actually harmful will show good results at first.

---

Mid-diastolic murmurs heard at the apex of the heart in certain cases of aortic regurgitation (distinct from Flint's murmur) of adherent pericardium and of mitral regurgitation, form the subject of a communication by Dr. Coombs to the *Bristol Medico-Chirurgical Journal.* It is pointed out that this particular strictly mid-diastolic and non-crescendo apical murmur is smooth and very variable in character, thus differing markedly from the typical bruit of mitral stenosis. Moreover, it is heard sometimes in patients proved by subsequent autopsy to be free from mitral stenosis. From the similarity of the murmur in the three classes of heart lesion mentioned, it is concluded that the causation is in all three the same, and is to be sought in some factor common to them all. Reasons are put forward for regarding the blood passing from auricle to ventricle before the auricular systole as the seat of production of this murmur. Now, great enlargement of the left ventricle is seen in all three of the lesions under discussion, and this enlargement is, of course, due to a combination of hypertrophy and dilatation. If, then, the dilatation were uniform, the cubic content of the dilated ventricle would vary as the cube of the linear dimension, whereas the area of the mitral orifice is enlarged only as the square of that figure. But in actual fact the disproportion between chamber and entrance is greater still, because the mitral ring, being less muscular and more fibrous than the rest of the wall of the ventricle, gives way less under strain or toxic influences. Dr. Coombs holds that the suction of the dilating ventricle in diastole draws blood through a comparatively narrow orifice, and so sets up the murmurs in question.

---

The same murmur is discussed also by Cole and Cecil in the *Johns Hopkins Hospital Bulletin.* These observers, like Dr. Coombs, emphasise the facts that the apical diastolic murmur of aortic regurgitation is neither rough, nor crescendo, nor limited to the region of the apex, and does not end in a short sharp first sound, as do both Flint's murmur and the presystolic murmur of mitral stenosis. It may at times, they say, be widely distributed throughout the axilla and is frequently best heard rather outside the actual position of the cardiac apex. On this point they differ from the English observer. In some cases there has been found no associated basal murmur, and the diagnosis has rested on the other signs of the case. These characteristics are to be found in both the arterio-sclerotic and the endocarditic types of aortic valvular disease; and the distribution of the bruits seems to be the same in each. The authors bring forward arguments to show that this murmur is identical with the usual basal murmur, which they find, as many others have noted, as common or commoner to the left of the sternum as to the right of it. Why it should be in certain cases louder at and outside the apex than at the base they expressly offer no theory; but they do not accept the suggestion put forward many years ago by Foster that the cause of the phenomenon is a lesion of the posterior cusp of the semilunar valves as opposed to the anterior two. They are at particular pains to anticipate the objection that the axillary murmur which they have been studying has anything to do with Flint's murmur, or with a mitral stenotic murmur. The ingenious hypothesis of Dr. Coombs had, naturally, not come under their notice when their paper was published, but the two contributions read side by side are most instructive.

---

The functions of the thyro-arytenoid muscles, and the exact manner in which the vocal pitch is determined, have been the subjects of various extremely divergent views. Some have maintained that the contraction of these muscles shortens the glottis or increases its tension, whereby the pitch of the voice is raised: others think that the effect is exactly the reverse, that the cords slacken when the muscles contract. Mr. J. D. Lithgow contributes a paper on this question to the *Journal of Laryngology,* in which he supports the latter view, holding that active contracture must cause the pitch to be lowered, and that passive lengthening by external forces has the opposite effect of raising the pitch. He has satisfied himself that the elastic vocal ligaments do not themselves determine the pitch, but follow the movements of the thyro-arytenoid muscles. Now it is proved, he says, that a contracted muscle is less elastic than a relaxed one; and also that the elasticity of passive muscle is increased by its extension. From this he concludes that the thyro-arytenoids have a dual *rôle* in the production of pitch. When passively stretched by the action of the crico-thyroid, the elasticity and pitch are raised, and when actively contracted both the latter are diminished. The upper vocal limit is reached when the crico-thyroid has stretched the (passive) thyro-arytenoid to its limit of elasticity; the lower when the latter has contracted as much as the former will permit of. Further, since a tired muscle becomes less elastic, the "flatness" of a tired singer is naturally explained. Also, the timbre of a note produced by contracture of the muscle will differ from that produced by its passive stretching, and will be richer in the lower harmonics.

THE theory of Meyer and Overton that the action of anæsthetics is dependent upon the power of their chemical substances to dissolve fats is already well known, and has been generally accepted. The especial action of these drugs upon the nervous system is explained by the preponderance of fatty substances in this part of the organism. In consequence it has occurred to Nerking to observe what modifications might result in the anæsthetic effects of these drugs by the intravenous injection of some lipoid such as lecithin. As a result of his experiments he has been able to demonstrate that when such injections of lecithin are made within a sufficiently short time after the animal is anæsthetised the anæsthesia is either curtailed or completely abolished. He states that the same effect results whatever drug is used to produce the anæsthesia. The assumption is, of course, that the anæsthetic is used up in combining with the lecithin circulating in the blood, and its effect on the lipoids of the organism is thus prevented.

---

AN interesting paper was read by Dr. Finkelstein before the Society of Medicine at Berlin on "Alimentary Fever." He maintains that a febrile condition dependent upon alimentary intoxication is a clinical entity, apart from any gastro-enteritis of microbic origin. Such intoxications have been attributed to toxins, ptomaines, abnormal fermentations, or putrefactions, but experimentation has failed to establish the presence of such causative agents. He points out the resemblance between these intoxications and diabetic coma—delirium, acetonuria, and sometimes even glycosuria. It has been supposed that in feeding with cow's milk it is the casein that is responsible for this morbid condition in infants. The author finds, however, that pure casein can be administered to dyspeptic infants with impunity. On the other hand, this alimentary intoxication may often be provoked by Liebig's bouillon, which contains 70 per cent. of carbohydrate. With dyspeptic children especially, excess of sugar will cause a rise of temperature, and with healthy children hypodermic injections of sugar have the same effect. A similar rise of temperature has been observed after injections of normal saline. The author suggests that under ordinary circumstances the intestine acts as a barrier to the introduction into the system of injurious substances, but that changes in the intestinal mucous membrane may break down this barrier, the discretionary power of the intestinal wall is lost, and the organism is no longer properly protected. Such cases of alimentary fever should always yield to dietetic measures, and, according to the author, actually do, the chief indication being the restriction of carbohydrates.

---

WITH the advent of antitoxin for diphtheria hopes were entertained that this discovery would be speedily followed by the elaboration of specific antitoxins for all other forms of bacterial infection. The fact that these hopes have not been realised is now explained on the supposition that, in the case of diphtheria and tetanus, for instance, the toxins of the specific germ are liberated into the blood plasma, and then give rise to the antigens—antitoxins, agglutinins, and bacteriolysins. Such toxins are known as exotoxins. In infections with the pneumococcus or meningococcus, on the other hand, the toxins are confined within the germ-cells—endotoxins; and the antitoxins, it is supposed, are also bound up within the bodies of the leucocytes. Acting upon this hypothesis, Hiss has experimented with the extracts of leucocytes from previously immunised animals, with the view that injections of such extracts should influence favourably the course of a specific infection. Nearly every animal thus treated recovered or was appreciably benefited, whereas every control animal died. In consequence of these experiments a trial with these extracts on human beings was considered justified, and a series of cases of pneumonia and meningitis has been treated in this way. In 22 severe cases of meningitis there was a mortality of 36.4 per cent. Seven cases of pneumonia were so treated, all of which recovered. It is stated in the report on these cases that a prompt amelioration of the subjective symptoms always followed the injections, together with a fall in temperature. The author admits that at present the method is still in the experimental stage and somewhat crude, but the results so far obtained appear to justify expectations of better things to come on the same lines.

---

IN a paper appearing in *La Semaine Médicale*, Dr. B. Schiassi, of Bologne, advocates the treatment of varicose veins in the legs by the intravenous injection of iodine solution into the internal saphenous vein. The idea of obliterating varicose veins by introducing some irritating substances within the vessels is of ancient origin, but the objection to the method is the fear of an embolus detaching from the thrombus thus formed. Dr. Schiassi argues, however, on theoretical lines, that such an accident is, under the circumstances, highly improbable, and considers that, in any case, his method sufficiently safeguards the patient. These are the chief details: Anæsthesia is obtained by spinal injection or by the local injection of novo-cocaine solution. An incision of 5 or 6 centimetres is made either above or below the condyle of the femur according to the case, and the saphena vein is exposed for the same length. The vein is ligatured at the upper angle of the wound, forceps are applied at the lower end, and the vein is divided near the ligature. A small glass tube with an olive-shaped end is introduced and tied in the free end of the vein, and this is attached to a glass syringe containing the iodine solution, 1 per cent. in distilled water, with 1½ per cent. of iodide of potassium. In this way 30 to 50 cubic centimetres of the solution are injected peripherally into the vein of the leg. The vein is then tied and resected and the wound sutured. A slight rise of temperature may follow the injection. About a week after the injection the superficial veins of the leg are completely obliterated and transformed into hard cords, the passive hyperæmia is abolished, and varicose ulcers, eczema, and other circulatory troubles are said to clear up.

